# Trophic ecology of *Octopus vulgaris* paralarvae along the Iberian Canary current eastern boundary upwelling system

**DOI:** 10.1038/s41598-023-35206-4

**Published:** 2023-05-30

**Authors:** Álvaro Roura, Stephen R. Doyle, Alexandra Castro-Bugallo, Nathan E. Hall, Ángel F. Gonzalez, Jan M. Strugnell

**Affiliations:** 1 La Trobe University, Melbourne, VIC 3086 Australia; 2 Ecology and Marine Resources, Instituto de Investigaciones Marinas (IIM-CSIC), Eduardo Cabello 6, 36208 Vigo, Spain; 3 Wellcome Sanger Institute, Hinxton, Cambridgeshire, CB10 1SA UK; 4 GMDx Group Ltd, Melbourne, VIC Australia; 5 Centre for Sustainable Tropical Fisheries and Aquaculture, James Cook University, Townsville, QLD 4810 Australia

**Keywords:** Marine biology, Next-generation sequencing, Molecular ecology

## Abstract

Our knowledge of the diet of wild octopus paralarvae, *Octopus vulgaris,* is restricted to the first 2 weeks of its planktonic phase when they are selective hunters found near the coastline. These small paralarvae, bearing only three suckers per arm, are transported by oceanic currents from the coast towards offshore waters, where they complete the planktonic phase over 2 months. Here, we have investigated the trophic ecology of *O. vulgaris* paralarvae in two contrasting upwelling sub-regions of the Iberian Canary current (ICC) eastern boundary upwelling system and have evaluated dietary change as paralarvae develop (inferred by counting the number of suckers per arm, ranging from three to 15) along the coastal-oceanic gradient during their planktonic phase. Using high-throughput amplicon sequencing, we have characterised the diet of 100 paralarvae collected along the Northwest Iberian Peninsula (n = 65, three to five suckers per arm) and off the west coast of Morocco (n = 35, three to 15 suckers per arm), identifying up to 87 different prey species. The diet of paralarvae varied along the ICC, with crabs (53.4%), siphonophores (12.2%), copepods (12.3%), cnidarians (8.4%) and pteropods (3.7%) accounting for 90% of the variability detected off NW Iberian Peninsula, whereas off W Morocco, crabs (46.2%), copepods (23.1%), cnidarians (12.9%), krill (9.3%) and fishes (4.2%) explained 95.6% of the variability observed using frequency of observance (FOO%) data. Ontogenetic changes in the diet based on groups of paralarvae with similar numbers per arm were evidenced by the decreasing contribution of coastal meroplankton and an increase in oceanic holoplankton, including siphonophores, copepods, pteropods and krill. Trophic niche breadth values ranged from 0.06 to 0.67, with averaged values ranging from 0.23 to 0.33 (generalist = 1 and specialist = 0), suggesting that *O. vulgaris* paralarvae are selective predators through their ontogenetic transition between coastal and oceanic environments.

## Introduction

Understanding trophic relationships during the early developmental stages of cephalopods (e.g. octopus, squids, cuttlefishes) is challenging for two primary reasons. Firstly, early life stage cephalopods are infrequently seen in zooplankton communities, i.e., rare occurrences with very low natural abundance^1^ normally expressed as individuals/1000 m^3^ or 10,000 m^3^ and, therefore, are challenging to collect. Secondly, because they quickly break down their prey, it is challenging to identify prey contents visually^1,2^. There are two modes of ingestion depending on the prey. Fish larvae are mechanically fragmented using the beak and radula and ingested in small pieces^1,2^, whereas crustacean prey are digested using a complex array of enzymes^3^, specifically evolved to remove the flesh from their exo- or endoskeletons. The beak and radula are then used to suck up the predigested prey leaving empty exoskeletons^4^, however, small pieces of prey can be occasionally ingested^5^. These ingestion strategies make studying stomach contents by traditional methods of morphological-based taxonomic identification difficult. There is only one such study on the diet of wild octopod paralarvae of the families Amphitretidae, Argonautidae and Octopodidae collected from the eastern Gulf of Mexico^1^, where prey fragments of euphausiids, fishes, non-cephalopod molluscs, chaetognaths, copepods, ostracods, decapods and hyperiid amphipods were detected.

In captivity, the common octopus, *Octopus vulgaris,* are active hunters immediately after hatching, armed with only three suckers on each arm^6^. Prey has been detected via analysis of DNA from partially digested and liquefied contents in captive *O. vulgaris* hatchings^7^, but the visual examination of the diet was precluded by the presence of amorphous material inside of the crop and stomach^8^. The first molecular attempt to unveil the diet of wild *O. vulgaris* paralarvae in the coastal area of Ría de Vigo, Northwest Spain, revealed up to 20 different prey species, showing that early hatchlings mainly preyed upon decapods, but also fish and euphausiids^8^. The prevalence of decapod families in the diet compared with the low abundance of these families in the zooplankton coastal communities^9^ suggested that *O. vulgaris* hatchlings were specialist predators, as shown by the low values of trophic niche breadth (Czekanowski’s Index, CI 0.04–0.37; generalist = 1 and specialist = 0)^10^. Further molecular studies, also carried out on early hatchings with three arm suckers over the continental shelf of northwest Spain, revealed up to 46 different prey species consisting of mostly decapods, but also included copepods, ophiuroids, bivalves, cladocerans, cnidarians, amphipods and chaetognaths^11^.

Our current understanding of the trophic ecology of *O. vulgaris* paralarvae is restricted to early stages with only three suckers on each arm^8,11^. Almost all *Octopus* paralarvae collected to date over the continental shelves of the northeastern Atlantic (n = 3254) had three suckers on each arm^10,12–16^. The only exceptions correspond to some *O. vulgaris* paralarvae collected in zooplankton tows in the English Channel^17,18^, later used for histological studies^19^. More recently, *O. vulgaris* paralarvae with more than three suckers per arm were collected along the Iberian Canary current (ICC) eastern boundary upwelling system, revealing that the paralarvae captured beyond the continental shelf (> 200 m depth) were older than those collected in the coastal areas with only three suckers^20^. Such dispersal suggested a coastal-oceanic life strategy for the planktonic phase of *O. vulgaris*^21^. During the planktonic phase beyond the continental shelf, additional suckers are added until 23–25 suckers are present on each arm, before returning to the coastal area to become benthic juveniles and adults^22^. Microbiome analysis of the paralarvae collected beyond the continental shelf showed a significant increase in bacterial diversity correlated with an increase in sucker number, which was hypothesised to be partly because of the change in the prokaryotic assemblage along the coast-ocean gradient^23^, and the ingestion of diverse prey in the oceanic areas^24^. However, the diet of paralarvae beyond the very early life stages and as they develop in the ocean and transition back to the continental shelf to mature remains broadly unknown.

Understanding the diet of the *O. vulgaris* paralarvae collected in the oceanic realm will help to unveil their trophic behaviour throughout their development in the plankton and provide important information regarding the nutritional requirements of *O. vulgaris*. Determining nutritional requirements that are critical for efficient growth and survival is invaluable for the development of commercial aquaculture^25,26^. Here, we have characterised the diet of different developmental stages of *O. vulgaris* paralarvae found in zooplankton samples along the ICC to evaluate (1) the differences in the diet in two contrasting upwelling sub-regions, NW Iberian Peninsula and W Morocco, and (2) the trophic behaviour of paralarvae as they travel along the coastal-oceanic gradient during the planktonic phase.

## Results

### Prey composition and abundance

Dissections of the digestive tract of 100 *O. vulgaris* paralarvae revealed that 91 had empty crops and nine stomachs with partially digested prey, none of which could be identified visually. Five paralarvae had no prey DNA detected; four were collected in Morocco (with three, four (n = 2) and six suckers per arm), and one was collected off the coast of Portugal with five suckers per arm. In total, 2,923,510 high-quality sequencing reads were obtained after filtering, with an average of 30,057 reads per paralarvae (ranging from 22 to 339,756 reads). Classification of the reads showed that 2,745,355 (93.9%) corresponded to *O. vulgaris*; 121,975 (4.17%) were identified as prey; and 56,180 (1.92%) were associated with contamination that was excluded from downstream analyses. Contaminants included fungi, humans, vertebrates, insects, as well as some marine species that were analysed in the laboratory in which the samples were processed, such as lobsters (see Appendix 1 for details). DNA of prey was detected in 95 out of 100 *O. vulgaris* paralarvae, with 1219 prey reads per paralarva on average (ranging from 1 to 29,677). The negative sample revealed 29 reads corresponding to *O. vulgaris* (Appendix 1).

**Figure 1:**
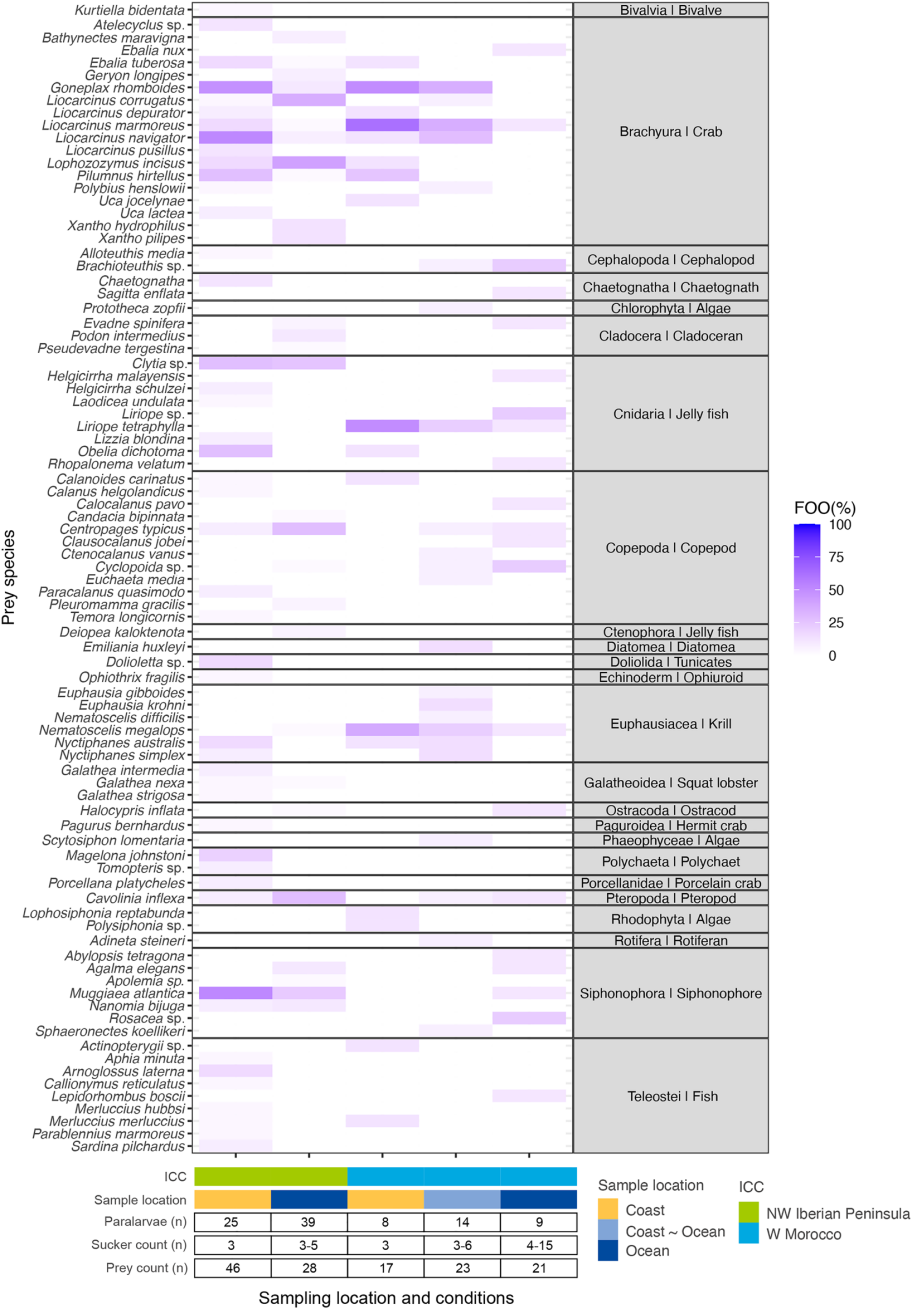
Prey detected in *Octopus vulgaris* paralarvae. Heatmap shows the frequency of observance (FOO %) of the prey detected in *O. vulgaris* paralarvae, grouped by the Iberian Canary current sub-regions sampled (ICC: NW Iberian Peninsula and W Morocco) and locations (coast, coast ~ ocean and ocean). Heatmap created with R (version 4.0.3).

**Figure 2:**
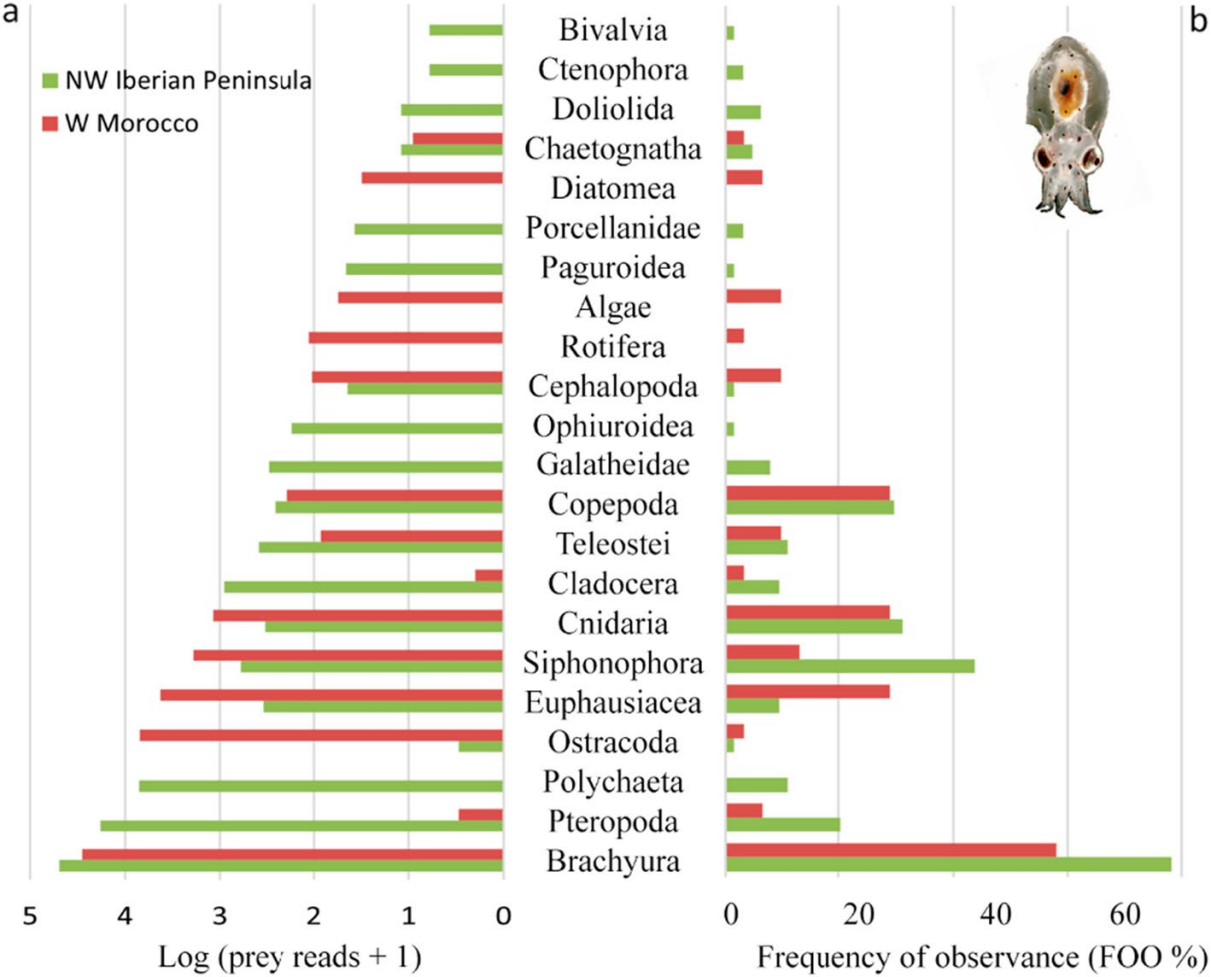
Main prey groups detected in the diet of *Octopus vulgaris* paralarvae in the two regions of the ICC sampled, NW Iberian Peninsula and W Morocco. (**a**) Number of reads log-transformed for each prey group ordered by the total number of reads. (**b**) Frequency of observance (%) of the different prey groups within the digestive tracts of *O. vulgaris* paralarvae.

Analysis of prey sequencing reads revealed 87 different taxa, 33 of which were exclusively detected near the coast, 36 from the ocean, and 18 in both environments (Fig. 1, table S1). An average of 3.6 prey were detected in each paralarva (ranging from one to 13 prey). The most common prey groups based on the number of sequencing reads were crabs (63.9%, 77,974 reads), pteropods (15.1%, 18,424 reads), polychaetes (5.9%, 7190 reads), ostracods (5.7%, 6980 reads), euphausiids (3.8%, 4594 reads) and siphonophores (2.1%, 2527 reads) (Fig. 1, see also Fig. 2a, with reads transformed as log (x + 1) to aid visualisation and comparison). However, read abundance contrasted with the importance of these prey groups in the diet. The most frequent prey group based on the frequency of occurrence (FOO; defined as the presence or absence of prey per sample, independent of the number of sequencing reads) in *O. vulgaris* paralarvae were crabs (71.6%, n = 68 paralarvae), siphonophores (32.6%, n = 31), cnidarians (31.6%, n = 30), copepods (29.5%, n = 28), euphausiids and pteropods (15.8%, n = 15), fish (10.5%, n = 10) and polychaetes and cladocerans (7.4%, n = 7) (Figs. 1, 2b).

The most abundant prey in terms of relative read abundance (RRA) across samples were the crabs *Goneplax rhomboides* (9.4%), *Lophozozymus incisus* (9.2%), *Liocarcinus navigator* (7.1%) and *Liocarcinus corrugatus* (6.8%) followed by the cnidarian *Liriope tetraphylla* (5.7%), the pteropod *Cavolinia inflexa* (5.5%) and the siphonophore *Muggiaea atlantica* (4.9%) (Appendix 2). The most frequent prey using FOO data were the crab *G. rhomboides* (detected in 26.3% of paralarvae), the siphonophore *M. atlantica* (24.2% of paralarvae), the crabs *L. navigator* and *L. incisus* (22.1% of paralarvae) and the cnidarian *Clytia* sp. (17.9% of paralarvae) (Fig. 1, Appendix 2).

### Diet along the Iberian Canary current upwelling system (ICC)

**Figure 3:**
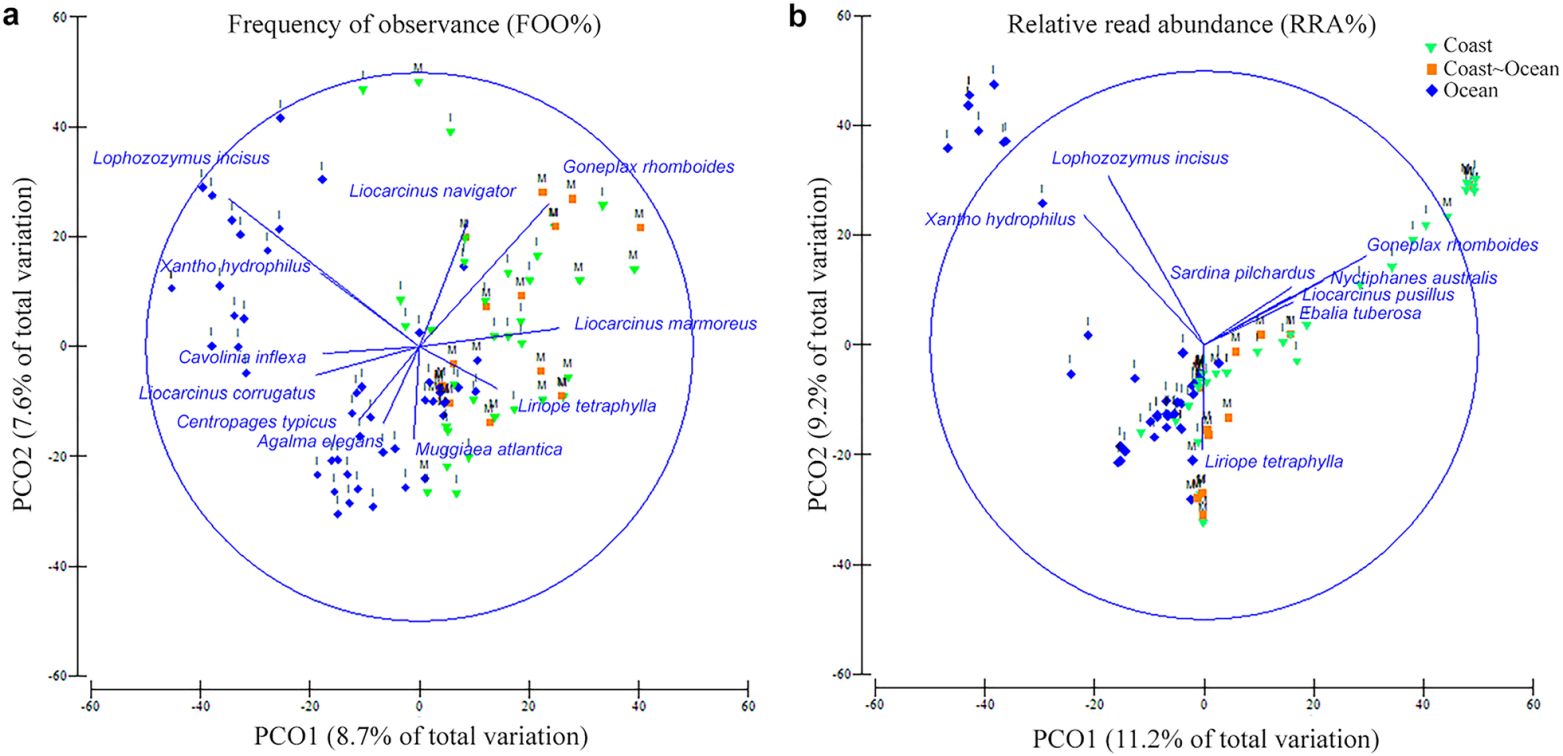
Principal coordinate analysis showing all *Octopus vulgaris* paralarvae according to their diets. Multidimensional representation of the diet of 95 *O. vulgaris* paralarvae based on the (**a**) frequency of observance (FOO) and (**b**) relative read abundance (RRA) data. The paralarvae are colour coded according to the different locations (C: coast in green, C~O: coast~ocean in orange, and O: ocean in blue), and letters indicating the ICC sub-regions sampled (I: Northwest Iberian Peninsula, M: Morocco). Blue vectors represent the prey with Pearson correlations > 0.35 with PCO axes 1 and 2.

Significant differences in the diet between the two sub-regions sampled within the ICC and also between the different locations sampled within those sub-regions, were identified using permutational multivariate analysis of variance (PERMANOVA; *p* < 0.001). Such diet differences can be observed in the PCO plots obtained with FOO (Fig. 3a) and RRA (Fig. 3b) data, where both factors ICC and location show similar patterns (covariation). The FOO plot shows great variability in the diets of the paralarvae and up to eleven species with correlations above 35% with PCO1 and PCO2 axes (Fig. 3a, blue vectors), with positive PCO1 values representing paralarvae collected in W Morocco and in the coastal location of NW Iberia, while negative values correspond with paralarvae collected from the ocean in NW Iberia. On the other hand, the RRA plot (Fig. 3b) shows lower variability in the diets and eight species with correlations above 35%, but with similar distribution to that obtained with FOO data concerning the ICC subregions. The gradient from the coastal (positive values) to the oceanic areas (negative values) is more evident in this plot. Given that the diets are significantly different in the two ICC sub-regions, a detailed description is provided per region.

### Northwest Iberian Peninsula

Sequencing of DNA from samples from the Northwest Iberian Peninsula resulted in a total of 1,850,867 sequencing reads classified as *Octopus* (1,765,671; 95.4%), prey (78,829; 4.3%), and contamination (6367; 0.3%, Appendix 1). An average of four prey (from one to 13) were detected in all octopus paralarvae except one sample with no prey DNA. Overall, 59 prey were identified: 35 crustaceans (16 crabs, seven copepods, three cladocerans, one hermit crab, three galatheids, one porcellanid, three euphausiids and one ostracod), seven fish, four siphonophores, four cnidarians, three molluscs (one pteropod, one cephalopod and one bivalve), two polychaetes, one ctenophore, one doliolid, one ophiuroid and a chaetognath (Fig. 1).

In terms of frequency of occurrence (Fig. 2b), crabs were the most common prey group detected (78.1%, 50/64 paralarvae), followed by siphonophores (43.8%, 28/64 paralarvae), cnidarians (31.3%, 20/64 paralarvae), copepods (29.7%, 19/64 paralarvae), pteropods (20.3%, 13/64 paralarvae), fish and polychaetes (10.9%, 7/64 paralarvae), and cladocerans and euphausiids (9.4%, 6/64 paralarvae) (Suppl. Table S1).

The diet of the paralarvae was significantly different according to the location collected (coast vs ocean, *p* = 0.001; factor correlated with PCO axis 1 in Fig. 3a,b) and the strata (*p* = 0.001). Pairwise analysis of the strata showed significant differences for the pairs < 5—< 100 m (*p* = 0.009) and < 5—< 500 m (*p* = 0.002). Pairwise analysis within the levels of the interaction factor (location and strata) revealed no significant differences.

**Figure 4:**
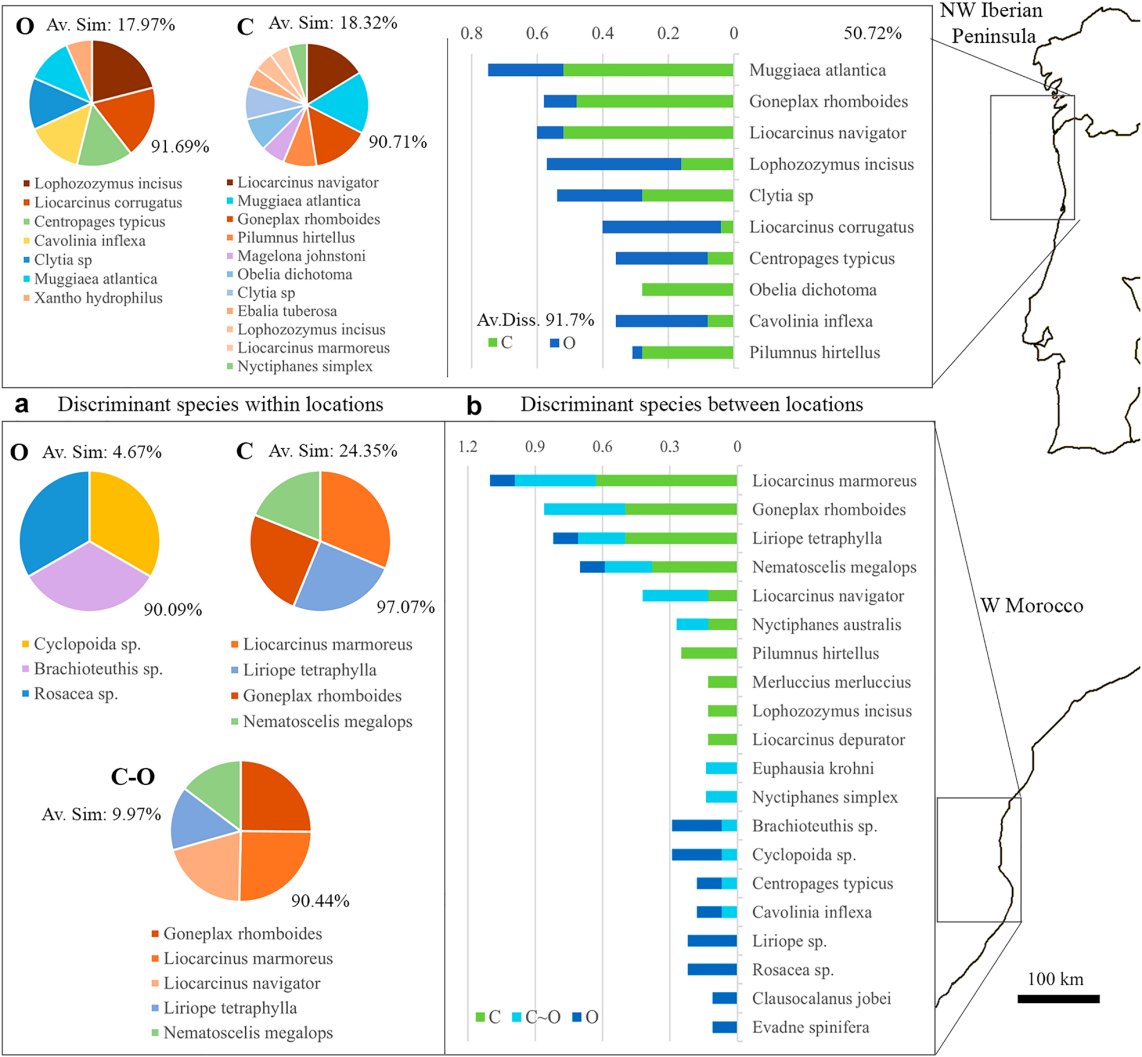
Geographical variation in the diet and discriminant prey species. (**a**) SIMPER analysis showing the averaged FOO of the discriminant prey (pie charts with the % of explained variance shown below the chart) that better describe the diets of *O. vulgaris* paralarvae collected at the different locations (C, coast; C-O, coast-ocean; O, ocean) in two sub-regions of the ICC upwelling ecosystem. Av. Sim. is the % similarity observed within the diets analysed at the different locations. (**b**) Column graphs show the average FOO of the ten most discriminant prey that better capture the differences between the locations. The numbers displayed on top of the species represent the % of variance explained by those species. Av. Diss. is the %  dissimilarity observed between the diets analysed at the different locations.

The most discriminant prey that defined the two locations sampled off Northwest Iberian Peninsula (coast and ocean) are represented as pie charts in Fig. 4a. The siphonophore *M. atlantica* and the crabs *L. navigator* and* G. rhomboides* were the prey that better described the diets of coastal paralarvae. In contrast, the crabs *L. incisus* and *L. corrugatus*, and the copepod *Centropages typicus* were the better descriptors of the oceanic paralarvae (Fig. 4a). Eleven species are listed in the coastal area accounting for 90.7% of the variability observed and seven species in the ocean account for 91.7% of the total variability. The diets were different at the two locations sampled, with only 18.3% and 18% of similarity in the coastal and oceanic areas, respectively (Fig. 4). The ten most discriminant organisms between the coastal and oceanic regions accounted for 50.7% of the total variability (Fig. 4b). The diet detected was markedly different as indicated by the 91.7% of dissimilarity observed between these two locations. DistLM results showed that 90% of the diet of the paralarvae collected along the Northwest Iberian Peninsula is predominantly represented by crabs (53.4%), followed by siphonophores (12.2%), copepods (12.3%), cnidarians (8.4%), and pteropods (3.7%) (Table 1).

**Table 1:** DistLM results for the analysis of the main prey groups (FOO data) contributing to the diet of *Octopus vulgaris* paralarvae in the ICC zones sampled.

Group	Indep. (%)	*p*-value	Adj. R^2^ (%)	SS (trace)	Pseudo-F	*p*-value	Prop. (%)	%
**Northwest Iberian Peninsula**								
Brachyura	53.4	**0.001**	37.6	144,000	3.3694	**0.001**	53.4	53.4
Siphonophores	16.3	**0.001**	49.6	32,786	3.7998	**0.001**	12.2	65.6
Copepods	19.3	**0.001**	60.2	33,205	2.4395	**0.001**	12.3	77.9
Cnidarians	16.2	**0.001**	71.2	22,584	3.6657	**0.001**	8.4	86.3
Pteropods	6.6	**0.001**	78.3	10,064	10.849	**0.001**	3.7	90.0
**Morocco**								
Brachyura	46.2	**0.001**	15.0	62,050	1.4829	**0.001**	46.2	46.2
Copepods	29.8	**0.001**	29.1	31,018	1.629	0.056	23.1	69.3
Cnidarians	24.6	**0.001**	46.5	17,322	2.4125	0.07	12.9	82.2
Euphausiids	22.4	**0.041**	63.5	12,498	2.55	0.083	9.3	91.5
Teleostei	9.4	0.725	73.8	5576	2.3788	0.159	4.2	95.6

The first two columns correspond to the marginal tests evaluating the independent contribution (Indep.) of the different prey groups. The following columns correspond to the sequential analysis of the groups retained by the model that maximised the % of explained variance (Adj. R^2^). Prop. Indicates the individual % of variance explained by the groups as they are added into the model. %. Indicates the cumulative % of variance explained as prey groups are added into the model.

Significant values are in bold.

### West Morocco

Sequencing data (total: 1,072,643 reads) from 35 paralarvae sampled off West Morocco revealed 979,684 reads corresponding to *Octopus* (91.3%), 43,146 to prey (4%), and 49,813 contamination (4.7%, Appendix 1). An average of 2.8 prey were detected from each paralarva (ranging from one to nine different prey), except for four samples with no prey reads. Overall, 45 prey were identified: 29 crustaceans (11 crabs, six euphausiids, seven copepods, one cladoceran and one ostracod), three fishes, five siphonophores, five cnidarians, two molluscs (one pteropod and one cephalopod) and one chaetognath (Fig. 1). The most frequent prey detected in the paralarvae were crabs (65.6% of prey reads, 18/31 paralarvae), cnidarians (2.7% of prey reads, 10/31 paralarvae), euphausiids and copepods (9.8 and 0.4% of prey reads, 9/31 paralarvae), and siphonophores, fish and non-octopus cephalopods (4.4, 0.2 and 0.2% of prey reads, 3/31 paralarvae) (Fig. 2b, Suppl. Table S1).

The diet of the paralarvae collected in Morocco showed significant differences according to the location sampled (*p* = 0.049, Fig. 3b). Pairwise analysis of the factor location revealed significant differences in diets for the pair coast—ocean (*p* = 0.005). SIMPER analysis revealed that only three to five prey species account for more than 90% (between 90.1% to 97.1%) of the variability detected within the three different locations sampled in Morocco (Fig. 4a). The best descriptor species for the coast and coast-ocean locations were almost identical (the crabs *L. marmoreus* and *G. rhomboides*, the cnidarian *L. tetraphylla* and the euphausiid *Nematoscelis megalops*), with the oceanic location being more different (cyclopoid copepod, *Brachioteuthis* sp. and the siphonophore *Rosacea* sp.). The diets detected at the three locations gained diversity as the paralarvae drift offshore from the coastal area (24.4% similarity) within the upwelling filament and its periphery (C–O samples, 10% similarity) into the open ocean (4.7% similarity, Fig. 4a).

The importance of the top ten species that define the changes between locations is better observed when their relative contribution is ordered by their decreasing importance within the coastal group. The diets of the paralarvae at the three locations had very little in common (Fig. 4b), except for the crab *L. marmoreus*, the cnidarian *L. tetraphylla* and the euphausiid *N. megalops*, whose contribution decreased from the coastal into the oceanic locations. DistLM results showed that 95.6% of the diet of the paralarvae collected off Morocco is represented by crabs (46.2%), followed by copepods (23.1%), cnidarians (12.9%), euphausiids (9.3%), and fishes (4.2%) (Table 1).

### Ontogenetic changes in diet: trophic behaviour

**Figure 5:**
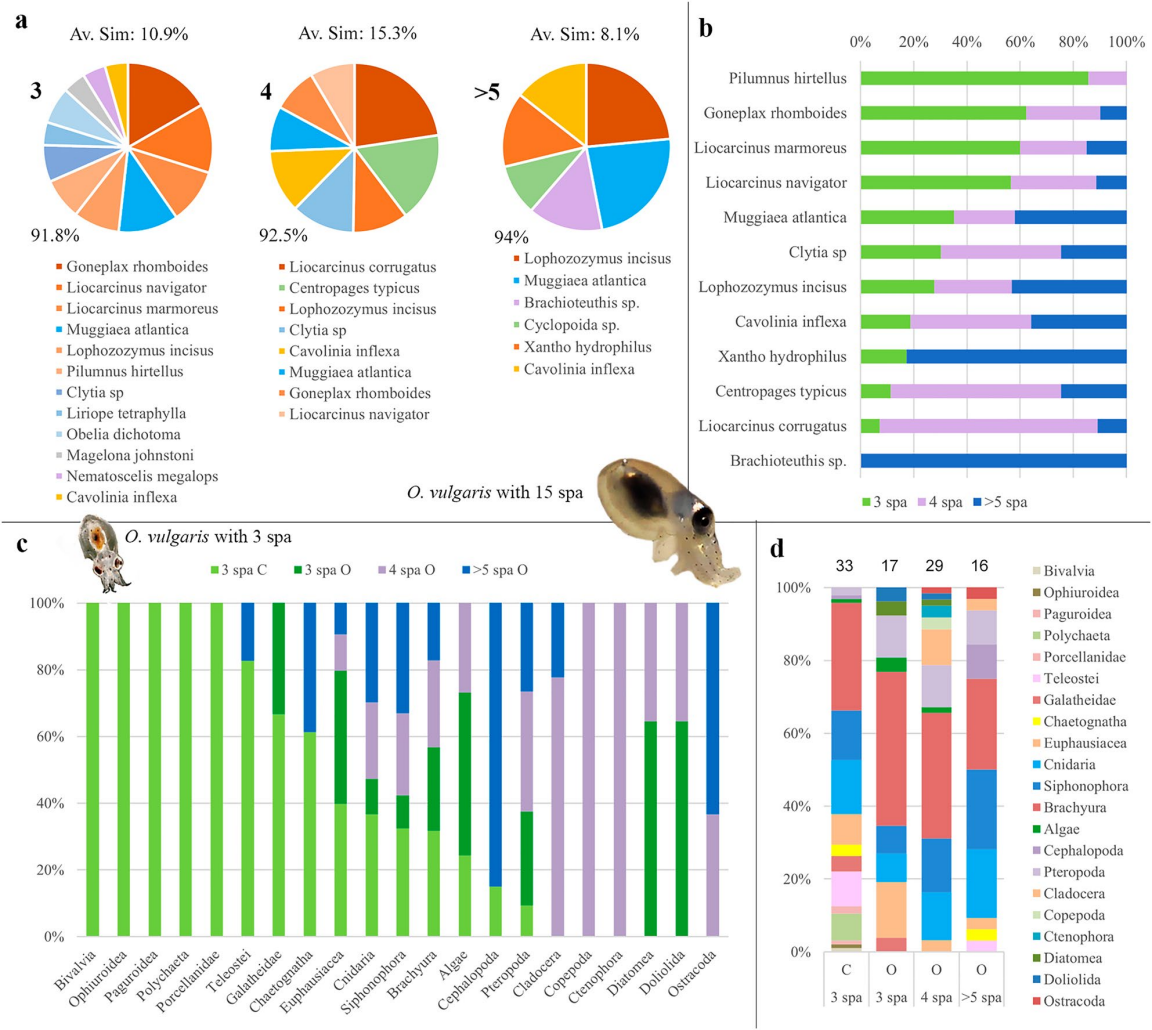
Ontogenic changes in the diet of *Octopus vulgaris* paralarvae using frequency of observance (FOO) data. (**a**) Comparisons of diet composition of samples with either three, four or more than five suckers per arm (spa) using SIMPER analysis. Pie charts show the % of variance explained by the top discriminant species that represent more than 90% of the diets of *O. vulgaris* paralarvae. (**b**) Relative contributions of the top ten discriminant species in paralarvae with different spa. (**c**) Prey groups detected in paralarvae with different spa but splitting the three suckers group into coastal (3 spa C) and ocean (3 spa O). (**d**) Relative contributions of prey groups through ontogeny. Numbers above the columns indicate the number of paralarvae. Abbreviations: Av. Sim. % of similarity observed within the diets analysed for each sucker group; C, coast; O, ocean.

Significant ontogenic differences between samples with a different number of suckers per arm (spa) was observed using FOO (%) data (PERMANOVA; *p* < 0.001). Pairwise analysis showed significant differences in the diet of paralarvae between 3 and 4 spa (*p* = 0.001), 3—> 5 (*p* = 0.004) and 4—> 5 suckers (*p* = 0.005). SIMPER analysis within the different groups of paralarvae revealed a decrease in prey diversity as the paralarvae grew. Pie charts in Fig. 5a show the species contributing > 90% of the total variability of the diet. Twelve species are responsible for 91.8% of the diet of paralarvae with three spa (collected from the coast and the ocean), whereas the diet of the paralarvae with four spa collected in the ocean is represented by eight prey (accounting for 92.5% of the variability), and six prey represent up to 94% of the diet of paralarvae with more than five spa. The ten most discriminant prey responsible for the differences detected between the diets of the three groups of suckers is shown in Table 2. The high dissimilarities obtained within each spa comparison (from 91.9 to 93.8%, Table 2) and the low similarities observed within each spa group (from 8.1 to 15.3%, Fig. 5a) suggest that the diets were very diverse.

**Table 2:** SIMPER analysis showing the FOO averaged abundance of the ten most discriminant species contributing to the ontogenic changes between paralarvae with different spa.

Species	3 spa	4 spa	Diss/SD	Var%
*Liocarcinus corrugatus*	0.04	0.45	0.79	7.53
*Goneplax rhomboides*	0.38	0.17	0.71	6.54
*Centropages typicus*	0.06	0.34	0.66	5.53
*Liocarcinus navigator*	0.3	0.17	0.62	5.58
*Lophozozymus incisus*	0.2	0.21	0.59	5.7
*Muggiaea atlantica*	0.26	0.17	0.58	4.83
*Liocarcinus marmoreus*	0.24	0.1	0.57	4.33
*Clytia* sp.	0.16	0.24	0.56	4.7
*Cavolinia inflexa*	0.1	0.24	0.55	4.54
*Pilumnus hirtellus*	0.18	0.03	0.46	2.52
Average dissimilarity = 92%				

Species	3 spa	> 5 spa	Diss/SD	Var%
*Muggiaea atlantica*	0.26	0.31	0.72	5.97
*Lophozozymus incisus*	0.2	0.31	0.69	6.36
*Goneplax rhomboides*	0.38	0.06	0.67	6.09
*Liocarcinus navigator*	0.3	0.06	0.56	4.84
*Liocarcinus marmoreus*	0.24	0.06	0.55	4.42
*Clytia* sp.	0.16	0.13	0.52	2.94
*Cavolinia inflexa*	0.1	0.19	0.49	3.73
*Xantho hydrophilus*	0.04	0.19	0.47	3.06
*Brachioteuthis sp.*	0	0.19	0.44	3.43
*Pilumnus hirtellus*	0.18	0	0.43	2.33
Average dissimilarity = 91.87%				

Species	4 spa	> 5 spa	Diss/SD	Var%
*Liocarcinus corrugatus*	0.45	0.06	0.81	8.4
*Centropages typicus*	0.34	0.13	0.7	6.57
*Lophozozymus incisus*	0.21	0.31	0.69	7.4
*Muggiaea atlantica*	0.17	0.31	0.65	6.61
*Cavolinia inflexa*	0.24	0.19	0.64	5.52
*Clytia* sp.	0.24	0.13	0.56	5.34
*Goneplax rhomboides*	0.17	0.06	0.47	3.33
*Liocarcinus navigator*	0.17	0.06	0.46	3.07
*Brachioteuthis sp.*	0	0.19	0.45	3.74
*Xantho hydrophilus*	0	0.19	0.45	2.69
Average dissimilarity = 93.77%				

The paralarvae were grouped into three groups (three suckers, 3 spa: n = 50; four suckers, 4 spa: n = 29; more than five suckers, > 5 spa: n = 16). Abbreviations: Diss/SD, discriminant power of each species; Var%, % of total variability explained by each species.

**Figure 6:**
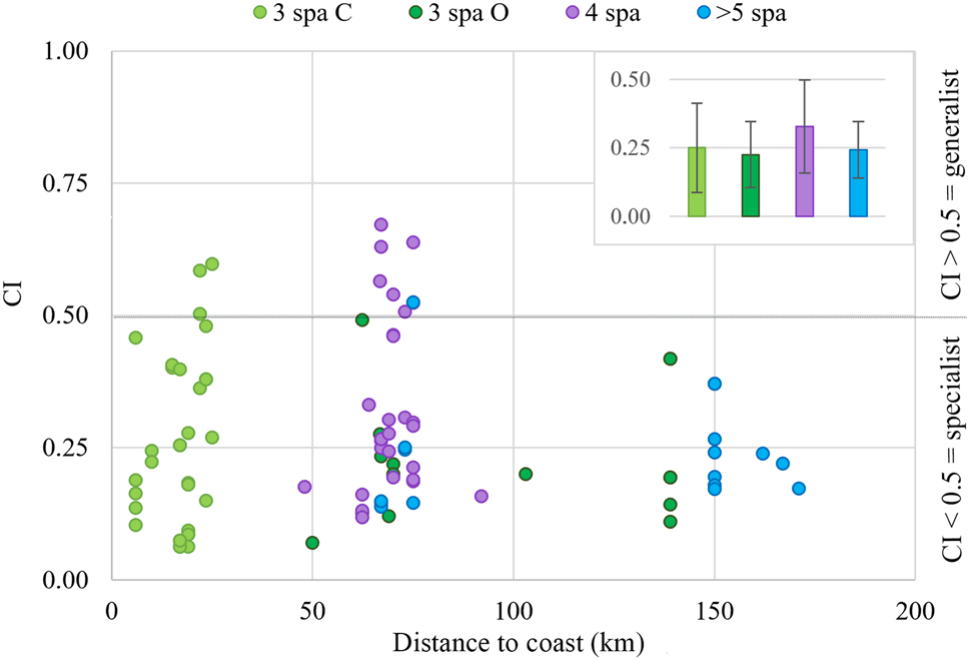
Ontogenetic changes in the trophic niche breadth (Czekanowski’s Index, CI) of *Octopus vulgaris* paralarvae. Trophic niche breadth (CI, ranges from 0 = specialist to 1 = generalist predator) was calculated for every *O. vulgaris* paralarvae and colour-coded according to the number of suckers per arm (spa). Averaged values and standard deviation for the different spa groups are shown in the upper right corner.

The importance of the top ten species that define the ontogenic changes is better observed when their relative contribution is ordered by their decreasing importance within the 3-spa group (Fig. 5b). It is evident that the contribution of meroplankton (i.e. larvae of benthic organisms like crabs) is replaced by holoplankton (copepods, siphonophores, cnidarians or pteropods) in older paralarvae. However, it is important to remember that the factors sucker number and location (coast, coast~ocean and ocean) are not independent. All paralarvae with 4 and > 5-spa were collected in the oceanic realm (that includes the coast-ocean and oceanic locations), while the paralarvae with 3-spa were the only ones collected in the coastal area but also in the two oceanic locations. Since the diet in these locations was significantly different (*p* = 0.001; Figs. 3a, 4), the 3-spa group was split into coastal (3 spa C) and oceanic (3 spa O) paralarvae (Figs. 5c,d, 6). This way, it is possible to visualise the ontogenetic changes from the coastal area into the open ocean and identify the main prey groups driving those differences. Overall, meroplankton groups like bivalves, ophiuroids, hermit crabs, polychaetes, and porcellanid crabs were restricted to the coastal area in recently hatched paralarvae (3-spa). As they drift into the ocean, new holoplankton groups (copepods, siphonophores, cnidarians, euphausiids, pteropods, cladocerans and ostracods) progressively become more frequent in the diet (Fig. 5c,d).

The linear index of food selection (L) was calculated for every prey group ingested at the different areas sampled (Table 3), considering the FOO within the digestive tracts and the natural abundances of the prey groups ingested (Suppl. Table S2). This index provides an idea of prey group selectivity by comparing the relative contribution of the prey groups in the plankton and their presence in the digestive tracts. Positive values were obtained for 22 out of 26 prey groups evaluated (in green, Table 3), showing a preference for these groups. Negative values were obtained for ophiuroids, euphausiids, siphonophores and the copepod family Centropagidae, indicating ingestion of prey groups that were abundant in the zooplankton samples of Ría de Vigo (ophiuroids) and specific samples of NW Iberian Peninsula (euphausiids, Centropagidae and siphonophores) and W Morocco (Temoridae and Centropagidae).

**Table 3:**
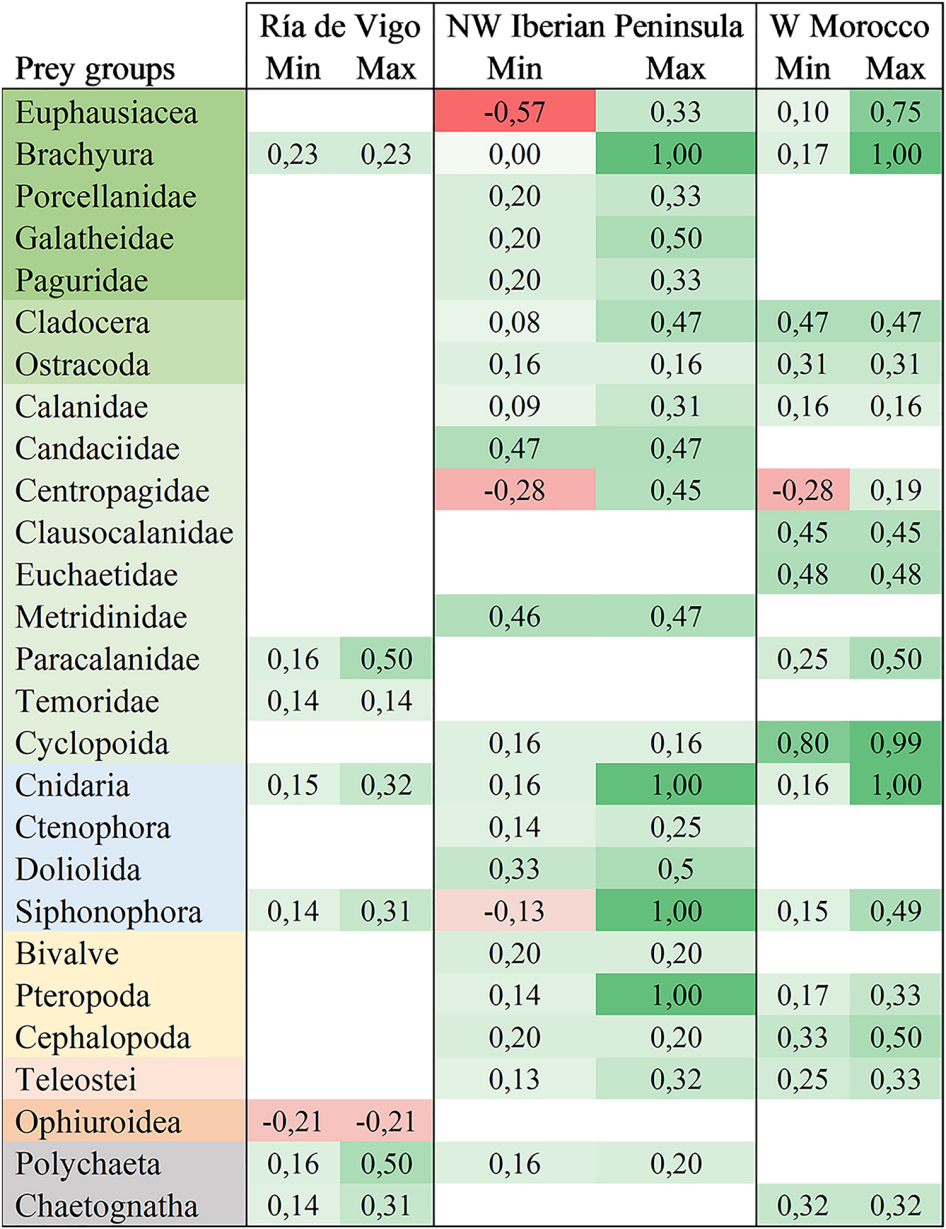
Maximum and minimum linear index of food selection values obtained for the different prey groups detected in *Octopus vulgaris* paralarvae along the ICC.

This index ranges from − 1 to + 1, with positive values indicating a preference (green colour), negative values indicating avoidance or inaccessibility (red colour), and zero values showing random feeding.

The other selectivity index analyzed, called trophic niche breadth, allowed the evaluation of paralarval diets by comparing the frequency of distributions (based on FOO data) of prey in the diet and the relative proportion of the prey in the zooplankton. This measurement, derived using the Czekanowski’s Index (CI), ranges from 0 to 1; in the context of diet, a value of 1 would indicate paralarvae target resources in proportion to their availability and reflects a generalist or non-specific predatory behaviour, whereas a value of 0 would indicate paralarvae are specialised exclusively on the rarest resource, and reflects a specialist predatory behaviour. The obtained CI ranged from 0.67 to 0.06, showing a broad range of trophic niches in the diets obtained for the paralarvae as they drift from the coast into the ocean (Fig. 6). Permanova analysis revealed no statistical differences in trophic niche breadth when paralarvae were grouped by sucker number (with averaged values of 0.25 ± 0.16 for 3-spa collected in the coastal area, 0.23 ± 0.12 for 3-spa collected in the ocean, 0.33 ± 0.17 for 4-spa and 0.24 ± 0.10 for > 5 suckers, Fig. 6 right corner). The averaged CI values suggest that *O. vulgaris* paralarvae can be considered specialist hunters with narrow trophic niches (CI < 0.5), feeding on prey groups that were not abundant in the plankton of the coastal and oceanic environments. However, within these groups, some paralarvae had broader trophic niches because they ingested prey that were abundant in specific samples (CI > 0.5), like euphausiids in sample S16 that accounted for 73% of the zooplankton abundance (Suppl. Table S2) or copepods of the family Centropagidae that dominated in most oceanic samples (Suppl. Table S2).

## Discussion

In this study, we present the first insights into the diet of *O. vulgaris* paralarvae during their planktonic phase beyond the continental shelf in two different sub-regions of the ICC. Our data reveal marked differences in their diet within and between different sub-regions and ontogenic changes throughout development. Such changes in the diet are related to changes in feeding behaviour but also to changes in the zooplankton communities along coastal-ocean gradients. The sampling sites range from a coastal embayment including the Ría de Vigo^9^, to the continental shelf of the Northwest Iberian Peninsula^27–30^ and the subtropical waters off Morocco^31–34^. These environments are markedly different in their physicochemical (temperature, salinity, transparency) and biological properties (primary production, zooplankton abundance and diversity), creating strong biological- and physical clines that paralarvae of *O. vulgaris* are subject to.

The seasonal upwelling filament of Cape Silleiro (Northwest Iberian Peninsula, 42–43° N) and the permanent upwelling filament located off Cape Ghir, Morocco (30–31° N) export approximately 4 × 10^8^ and 31 × 10^8^ kg/year of organic carbon to the adjacent shelf representing 20 and 60% of the total phytoplankton primary production in these regions, respectively^35^. The upwelling filaments disrupt the strongly stratified oceanic waters creating distinct community gradients that contrast with the open ocean communities^36^. *Octopus vulgaris* paralarvae hatch in coastal areas and are transported by upwelling filaments hundreds of kilometres offshore to complete their planktonic phase^21^. As the paralarvae drift with the currents from the coast into the ocean, they are surrounded by zooplankton communities that gradually become more diverse but less abundant. In the oceanic environment beyond the continental shelf, 58 prey species were detected (39 being exclusive of this environment, Suppl. Table S1), including pteropods, ostracods, ctenophores, doliolids and oceanic cephalopods that were not detected before in *Octopus* gut contents.

Previous research suggests that the faunal composition in the ocean and upwelled waters of the ICC is dominated by small and medium-size copepods (accounting for 82.5–87% during the annual cycle)^36^. Despite being the dominant taxa in the zooplankton, copepods were not the most prevalent group in the diet of *O. vulgaris* paralarvae, being detected in less than one-third of the paralarvae sampled in the Northwest Iberian Peninsula and Morocco (29.7% and 9% respectively), and only representing 0.4 and 0.3% of RRA. The present data, together with the low trophic niche breadth values (Fig. 6) and the positive linear indices or prey selection (Table 3), suggest that the paralarvae do not capture prey opportunistically based on their abundance (as expected for a generalist predator), but rather on other prey characteristics that can be related to their escape response or nutritional aspects. Copepods are fast swimmers with erratic movements that are difficult to predict and represent challenging prey for *O. vulgaris*. Copepod capture is a skill acquired throughout the planktonic stage, as observed with the squid *Loligo opalescens* in captivity^37^. Apart from prey characteristics, the anatomy of octopus paralarvae also limits their ability for prey hunting. Cephalopod paralarvae with a pair of feeding tentacles, such as loliginids or ommastrephids^1,2,11,38–40^, are better copepod hunters than *O. vulgaris* paralarvae which do not possess feeding tentacles. Herein, we observed that the paralarvae collected in the ocean consumed more copepods than those in the coastal area (Fig. 1, Suppl. Table 1). Since the paralarvae found in the open ocean are older (and have longer arms with an increased number of suckers) than those found near the coast^20^, this increase in copepod predation suggests that the paralarvae hunting skills increase as they develop, as it was observed for squid paralarvae^36^.

The diets analysed showed that the diversity of prey is slightly higher in the oceanic area (57 different prey species detected in 62 paralarvae) than in the coastal area (51 different prey species detected in 33 paralarvae). However, this pattern is not consistent along the ICC: in the coastal and oceanic regions of the NW Iberian Peninsula, 46 and 28 prey species were detected in 25 and 39 paralarvae, respectively (Fig. 1). Conversely, 17 and 44 prey species were detected in eight and 23 paralarvae of Western Morocco's coastal and oceanic areas. The overall prey diversity detected in the coastal area is higher than previous studies, where 46 different prey taxa were detected in 56 out of 64 *O. vulgaris* with three suckers per arm with the COI gene^11^. Prey detected were very similar, except for the presence of polychaetes, cephalopods, doliolids and ctenophores, detected for the first time in this work. It is important to mention the absence of decapod families including Processidae, Alpheidae, Crangonidae and Thalassinidae, formerly detected in other dietary studies in *O. vulgaris*^8,11^ but not present in this work. One possibility is that the modified primers, which included inosines to bind to any of the four nucleotides rather than degenerated bases^41,42^, may have prevented the amplification of those families.

The identification of discriminant species for the spatial and ontogenetic analyses carried out in this work was based on FOO rather than RRA. It is often assumed that because conversion to occurrence data moderates the impact of taxa-specific bias in marker signal, it provides a conservative view of diet. While it is true that occurrence-based summaries of diet are less affected by recovery bias and not skewed by read number, it has been observed that it is not necessarily true that they provide a more accurate representation of overall diet^43^. To evaluate this, we compared the discriminant species that defined the diets of the paralarvae at the different locations within the two areas samples of the ICC, using different datasets: FOO, RRA and log (x + 1) (Suppl. Fig. S1). FOO-based analysis recovered more discriminant species than log-based or RRA-based analyses, the latter being the most affected by read number. As an example, the coastal location of the NW Iberian Peninsula is defined by 10 species using FOO, seven species with log (x + 1) and five species with RRA. The primary drawback of occurrence data sets is that the importance of rare food taxa is often artificially inflated at the expense of food taxa eaten in large amounts^42^. However, there is a good concordance between the FOO and log (x + 1) analyses that identified similar discriminative power and explained variance for the top discriminant species. Contrarily, RRA-based analysis showed a reduced number of species with increased discriminatory power and explained variance due to the high read numbers of certain species.

The high diversity of euphausiids, cnidarians and siphonophores detected as prey in the ocean was remarkable, with siphonophores and cnidarians being the second and third most frequent prey ingested by *O. vulgaris* paralarvae (Fig. 2b). Natural contribution of gelatinous zooplankton like cnidarians and siphonophores is important inside the upwelling filaments, and their abundance gradually decreases as these mesoscale structures venture into the open ocean^44^. This trend was detected for the cnidarian *Liriope tetraphylla* in W Morocco, where a strong upwelling filament was sampled. *Liriope* was one of the top discriminant species defining the three locations sampled (Fig. 4a), with a relative contribution to the diet that decreased towards the oceanic samples (Fig. 4b).

In coastal areas, gelatinous zooplankton have also been detected as prey in *O. vulgaris* with three suckers^11^ and loliginid paralarvae, like *Alloteuthis media* and *Loligo vulgaris*^11,38^. Different facts might help to explain both the predation and the importance of these organisms in cephalopod paralarvae. These organisms are protein-rich (18.3 ± 7.8% of dry mass^45^), thus providing an easily digestible source of soluble nutrients^45,46^. Compared with highly motile prey like copepods, gelatinous plankton are relatively large slow drifters with predictable movements that are easy to capture by planktonic predators like cephalopod paralarvae^11,38^, fish larvae^47^ and crustaceans^48,49^. According to the optimal foraging theory, the paralarvae will adopt a foraging strategy that provides the most benefit for the lowest cost, maximising the net energy gained. Within that scenario, a large gelatinous prey with lower energetic value would be easier to subdue than a more vigorous energy-rich prey (e.g. copepods), saving energy. Alternatively, considering the energy gained from the secondary copepod prey vs primary gelatinous prey, perhaps the octopus is specifically targeting the copepod rather than the gelatinous organism. Another important aspect of ingesting gelatinous prey is that transparency plays a key role in the open ocean for epipelagic organisms. This strategy is the most common solution to the dilemma of having nowhere to hide, a trait present in almost all marine phyla (e.g. salpids, crustaceans, leptocephalus, heteropods, polychaetes, chaetognaths or ctenophores, among others) inhabiting the epipelagic realm. *Octopus* paralarvae are completely transparent except for membranes enclosing the eyes and digestive gland, which are covered by reflective cells called iridophores that act as an ambient light reflector, concealing the opaque body organs^22^. The ingestion of transparent prey tend to make the paralarvae less conspicuous and more difficult to target by predators. This strategy of transparent predators ingesting transparent prey has also been suggested for the European eel larvae^50^ and lobster larvae^45,48^.

Crabs were the most common prey in all octopus analysed (68 out of 95 paralarvae analysed), even though their abundances in the ocean are an order of magnitude less (Table S2) than in shelf communities^9,10,33^. This crustacean group was preferentially ingested in all areas analysed (Table 3, green colour), and some of the species detected were among the most discriminant species to define both the spatial (Fig. 4a,b) and ontogenic variability in the diet (Fig. 5a,b). Interestingly, different crab larvae were consistently detected in paralarvae collected in both areas of the ICC, like *Goneplax rhomboides or Liocarcinus navigator* (Fig. 4a,b). These two species were mainly associated with coastal locations in both areas but also present within the coastal~ocean locations owed to the offshore transport within the upwelling filament. Most larvae cannot swim against horizontal currents but can swim faster than vertical currents to maintain a preferred depth, thus limiting their cross-shore dispersal^51^. Studies of larvae dispersal in the ICC suggest that vertical behaviour is one of the main biological mechanisms for crustacean larval retention over the shelf^28,29^, fish larvae retention^52^ or fish larvae dispersal^53,54^. Upwelling filaments transport phytoplankton and zooplankton biomass between the productive coastal area and the oligotrophic oceanic realm^29,30,35^. Crab larvae are diel vertical migrators, and their horizontal distribution in the plankton (alongshore vs offshore)^28^ greatly depends on their vertical distribution in the water^55^. Considering the crab species ingested, and their location (coast vs ocean), it is evident that the upwelling filaments also drifted some crab larvae into the open ocean, as it occurred with *O. vulgaris*^21^. Some of these species are *Lophozozymus incisus* or *Liocarcinus corrugatus* in the NW Iberian Peninsula, as well as *Liocarcinus marmoreus, L. navigator* or *G. rhomboides* in W Morocco.

To our knowledge, it is the first time that the squid, *Alloteuthis media* has been detected as prey in *O. vulgaris* paralarvae; only one paralarva in the coastal region of the Iberian Peninsula was positive for *A. media*, where it is the main loliginid paralarvae present in the zooplankton^56,57^. Similarly, *Alloteuthis subulata*, *O. vulgaris* and the octopod *Eledone cirrhosa* have been detected as prey of *Loligo vulgaris* paralarvae collected in the Northwest Iberian Peninsula^38^. An undescribed *Brachioteuthis* species (99.1% homology with sequence MT223356, Appendix 1) was also detected in three *O. vulgaris* paralarvae with > 5 spa collected in the oceanic area off Morocco, where *Brachioteuthis riisei* was one of the most abundant cephalopod paralarvae sampled^21^. The methodology applied in this work to characterise the diet prevents the detection of predation in conspecifics, but it may also constitute a potential prey since cannibalism has been detected in *Octopus* species^58,59^.

This study did not attempt to separate primary versus secondary prey items; items designated as prey could contribute either way. However, specific taxa detected in the digestive tracts of the paralarvae like rotifers, diatoms, or algae might result from secondary predation^60^. Considering *Octopus* paralarvae are visual predators that attack large prey compared with their size^22^, these groups are too small (rotifers or phytoplankton) or undigestible (algae) to be captured by the paralarvae. It is possible that these groups were ingested by other prey, like copepods or decapod larvae, shortly before these were captured by the paralarvae. The longer a prey item is inside a predator’s gut, the harder it is to detect it because of DNA degradation. If the prey ingested by the paralarvae also ingested a prey item shortly before being captured, it could be possible to detect the DNA of both organisms inside the predator^61^. Molecular techniques are powerful tools to unravel trophic links, even in small organisms like *O. vulgaris* paralarvae. When dissections were carried out, only 9% of the paralarvae had amorphous contents inside the crop and stomach. However, prey DNA was successfully detected in 95%, highlighting the importance of molecular studies even when no prey contents are present.

Current aquaculture practices of octopus suffer from high levels of mortality, which may in part be due to sub-optimal diets in captivity^26^. These diets are based primarily on *Artemia* spp. (commonly used in aquaculture) enriched with commercial products or supplemented with other organisms like crustacean zoeae, copepods or amphipods. The increasing diversity of bacterial families detected in wild *O. vulgaris* paralarvae^24^*,* compared to the decreasing bacterial richness obtained in captive *O. vulgaris*, was hypothesised to be related to prey diversity available in the open ocean^24^. Our work shows that *O. vulgaris* paralarvae ingest numerous prey during the planktonic phase (at least 87 different taxa). Each prey has its own microbiome, thus adding complexity to the paralarvae’s microbiome. This natural prey diversity in the wild heavily contrasts with the mono-diets commonly used in aquaculture, which offer less enriched nutrition and poor microbial communities, even though recently hatched octopus paralarvae possess a diverse microbial community^24^. However, diets based on *Artemia* profoundly impact the microbiota after a few days, leading to the expansion of opportunistic and pathogenic populations^24^. This has also been observed in other organisms reared in captivity, such as olive flounder^62^ or white shrimp^63^. The *O. vulgaris* paralarvae studied in this work provide unique insight into their trophic ecology during the planktonic phase in the oceanic realm. This information can provide new avenues of research in captivity to reduce the high mortality levels currently constraining octopus aquaculture.

## Material and methods

### Oceanographic context

**Figure 7:**
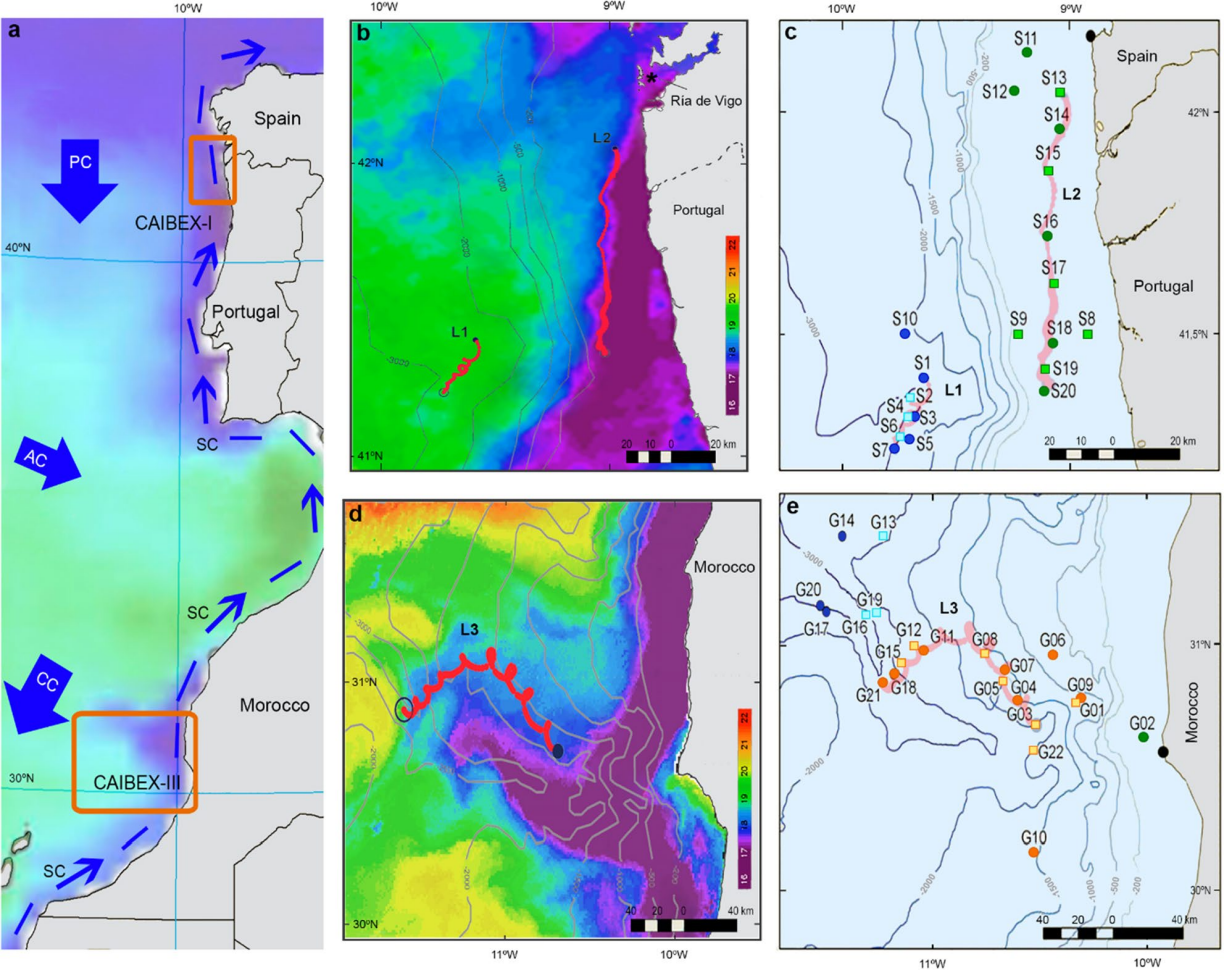
Sampling area, Lagrangian buoys and zooplankton tows. (**a**) Iberian-Canary current eastern upwelling system showing the sub-regions sampled (orange boxes) and the main currents (AC, Azores Current; CC, Canary current; PC, Portugal Current; SC, Slope current). (**b**) Trajectory of the buoys during the first (L1) and second Lagrangian experiments (L2) in Iberian waters overlaid on sea surface temperature (SST) at the end of L2 (July 20th). The asterisk shows the location inside the Ría de Vigo where *O. vulgaris* paralarvae were collected on September 22nd 2010. (**c**) Zooplankton samples were collected during CAIBEX-I off the Northwest coast of the Iberian Peninsula. Samples S1–S7 correspond to L1, carried out in the open ocean (blue), and S13–S20 to L2, carried out over the continental shelf (green). (**d**) The trajectory of the buoy during the third Lagrangian experiment in Moroccan waters (L3) overlaid on SST at the beginning of the experiment (August 24th). (**e**) Zooplankton samples collected during CAIBEX-III over the continental shelf off Morocco (green), following the upwelling filament during L3 (red), in the area affected by the upwelled water over the continental slope (orange) and in the open ocean (blue). In (**c**) and (**e**), the light squares and dark circles represent samples that were taken either during the day or night, respectively. Maps created with R (version 4.0.3; https://www.r-project.org/).

The Iberian–Canary current eastern boundary upwelling system (ICC) constitutes one of the world's oceans' four main eastern boundary upwelling systems. The ICC covers the latitudinal range between 12° and 43° N and can be broadly divided into five sub-regions according to their biogeographical characteristics^64^. This work is centred in three of these five sub-regions: the Galician, Portuguese and the Moroccan sub-regions (Fig. 7a). The NW Iberian Peninsula sub-region is the northernmost part of the ICC and includes the Galician and Portuguese sub-regions that are characterised by seasonal upwelling. During spring and summer (from March–April to September–October), north-easterly winds predominate in the Iberian basin, and mesoscale upwelling filaments develop intermittently (Fig. 7b) in association with irregularities in the coastline like capes^65–67^. On the other hand, the Moroccan sub-region experiences year-round upwelling that vary seasonally, with extended upwelling filaments (Fig. 7d), absence of freshwater inputs and massive dust inputs from the adjacent Sahara Desert^64,68^.

### Zooplankton sampling

Plankton samples were collected during the multidisciplinary project “Canaries-Iberian Marine Ecosystem Exchanges (CAIBEX)” off the coast of Northwest Iberian Peninsula (CAIBEX-I, June 7th–24th) and Cape Ghir, West Morocco (CAIBEX-III, August 16th–September 5th) in 2009 onboard the research vessel (RV) “Sarmiento de Gamboa” (Fig. 7a). Two lagrangian experiments that followed two different water masses identified with drifting buoys were carried out off NW Iberian Peninsula: (1) an upwelling-relaxation event in the open ocean in front of Portugal (L1: July 10th–13th inclusive Fig. 7b, samples S1–S7 in blue Fig. 7c); and (2) an upwelling event with alongshore transport along the continental shelf of Spain and Portugal, that can be identified by the sea surface temperature (SST) obtained at the end of the experiment with the violet colours representing the cold upwelled waters (L2: July 17th–20th inclusive, Fig. 7b, samples S13–S20 in green, Fig. 7c). Between L1 and L2, zooplankton samples were collected following a coastal-ocean gradient off the Portuguese coast (S8-S10) to observe the changes between these two environments, as well as two samples from the continental shelf of Galicia (S11 and S12). In contrast, a single Lagrangian experiment was carried out off the coast of W Morocco (31° N) following a strong upwelling filament^69,70^ forced by the prevailing trade winds from the coast into the open ocean (L3: August 23rd–31st inclusive Fig. 7d, samples in orange, Fig. 7e). The magnitude of the cold upwelling filament was visible from the SST obtained from satellite data, as shown in Fig. 7d. Samples were also collected over the continental shelf (in green, Fig. 7e), in an area affected by the upwelled water over the continental slope (in orange, Fig. 7e), and in the open ocean (in blue, Fig. 7e).

Mesozooplankton samples were collected day and night with two 750 mm diameter bongo nets equipped with 375 µm mesh and a mechanical flow meter. Three double-oblique towings were carried out at a ship speed of 2.5 knots over the continental slope (> 200 m depth): (1) from 0 to 500 m (< 500), (2) from 0 to 100 m (< 100), and (3) at the surface from 0 to 5 m (< 5). Over the continental shelf (< 200 m depth), only two double oblique towings were collected: from the surface down to 100 m (< 100 m, when the sea bottom was less than 100 m, the bongo net was lowered 10 m above the bottom) and at the surface (< 5). The bongo net was first lowered to the desired depth, towed for 30 min and subsequently hauled at 0.5 m s^–1^. The net was recovered, cleaned on board, and placed back into the sea for the next towing. Plankton samples were fixed with 96% ethanol and stored at − 20 °C to facilitate DNA preservation. Mesozooplankton abundance was estimated for each sample by subsampling the original sample into an amount suitable for examination using a Folsom splitter. The subsample was made up to 300 ml, and several aliquots of 3 ml were obtained with a Stempel pipette, then identified and counted until at least 500 individuals were enumerated. Organisms were identified using a binocular (Nikon SMZ800) or inverted microscope (Nikon Eclipse TS100) to the lowest taxonomic level.

All cephalopod paralarvae were separated from the zooplankton samples in the laboratory and stored individually in 70% ethanol at − 20 °C. In total, 134 *O. vulgaris* paralarvae were collected in NW Iberian Peninsula (n = 99) and W Morocco (n = 35), after which the number of suckers on the arms was counted. Of these, 60 paralarvae were chosen from the NW Iberian Peninsula (ranging from three to five suckers per arm) and 35 from Morocco (ranging from three to 15 suckers per arm) to study their diet (Table 4). Additionally, five recently hatched *O. vulgaris* paralarvae collected onboard the RV “Mytilus” at night at the surface of the Ría de Vigo (September 22nd 2010) were also included in the NW Iberian Peninsula group (n = 65). More detail of the sampling in the Ría de Vigo in 2010 can be found in^10^.

**Table 4:** The number of *Octopus vulgaris* paralarvae (n) collected during the different surveys and those analysed for diet (d) along the Iberian Canary current (ICC) eastern boundary upwelling system.

Survey	ICC	Location	n	d	Suckers	Depth (m)	Distance (km)
Ría de Vigo 2010	Northwest Iberian Peninsula	Coast	209	5	3	30–115	5–10
CAIBEX-I	Northwest Iberian Peninsula	Coast	51	20	3	62–147	10–31
		Ocean	48	40	3–5	1940–3105	62–75
CAIBEX-III	West Morocco	Coast	9	9	3	88–90	19
		Coast~Ocean	14	14	3–6	787–2720	48–162
		Ocean	12	12	4–15	2418–3110	140–171

The distance in km refers to the horizontal length from the collection sites to the coastline.

### Library preparation and sequencing

The digestive tract of *O. vulgaris* paralarvae, which included the oesophagus, crop, stomach, caecum, digestive gland, and intestine, was dissected using entomological needles rinsed in ethanol and burned between every dissection. DNA was extracted using the QIAGEN DNeasy Blood and Tissue Kit, according to the manufacturer’s instructions. A slight modification was made at the final elution stage of the extraction to increase the yield; the elution was repeated twice using two 20 µL aliquots of 45 °C ultrapure water and stored as a combined 40 µL eluate before use. A 300 bp region of the mitochondrial COI gene was amplified with NICO-F and NICO-R primers. The designed primers NICO-F and NICO-R (Suppl. Table S3) are a modification of the degenerated primer mICOIintF^71^ and HCO^72^, respectively. These primers were modified to add an inosine (I) that complements all four nucleotides^42^ to capture a greater fraction of the prey. The primers contained a 5’ tail containing a universal Illumina adapter sequence (italics) to enable multiplex indexing via primer extension PCR (Suppl. Table S3).

PCR reactions (12.5 μL) contained 10–30 ng of DNA template, 6.25 μL of RedTAQ ReadyMix (Sigma-Aldrich), 0.35 μL NICO-F (10 μM), 0.2 μL NICO-R (10 μM) and 0.1 μL MgCl_2_ (25 nM). Touchdown-PCR was used to facilitate primer attachment with targets that were not 100% complementary. Cycling conditions consisted of an initial denaturation at 95 °C for 3 min followed by ten cycles of denaturation at 95 °C for 30 s, annealing at 57 °C for 30 s—decreasing 1 °C per cycle—and an extension at 72 °C for 40 s, then 25 cycles of 95 °C for 30 s, annealing at 47 °C for 30 s and an extension at 72 °C for 40 s, with a final step at 72 °C 4 min. PCR products were cleaned with AMPure beads according to the manufacturer’s instructions and quantified with a Qubit HS kit in a Qubit™ fluorometer. Illumina multiplex indexes were added to the sequences in a second-round PCR before pooling 0.5 ng of each PCR product in a meta sample at a final concentration of 2.5 nM. To do so, some samples had to be diluted while others were repeated in duplicates and then concentrated to reach the same amount of DNA per sample. After adding 10% PhiX and diluting to 12.5 pM, the pooled library was sequenced using a v3 2 × 300 bp sequencing kit on an Illumina MiSeq. DNA extractions and PCR reactions were conducted in different rooms to avoid cross-contamination. Negatives were included for each round of PCR (with miliQ water instead of DNA) and in the meta-sample for sequencing.

### Quality filtering and bioinformatic analyses

Quality filtering was carried out following recommendations for Illumina platforms^73^. Reads that did not meet the following standards were removed: (1) Phred score below 30 (i.e., one error in 1000 bases), (2) less than 75% of target length, (3) less than three consecutive low-quality calls, (4) reads with ambiguous calls and (5) reads with less than five identical copies. The remaining paired-end reads were merged with PEAR v0.9.4^74^ using a 99% sequence similarity threshold (minimum of five sequences per cluster). Merged reads were demultiplexed into individual sample read sets based on their corresponding adapter combination. Reads for which the indexes/primers did not match the expected sequences were discarded. The remaining reads were then filtered against a Kraken (v0.10.4) database to exclude archaeal and viral contamination^75^. The UCHIME algorithm of USEARCH (v 6.0.307)^76^ was used to check for and remove chimeric sequences. Consensus sequences were BLASTed against sequences on GenBank, with the BLASTn function, and those with similarities above 80% were recovered. Amplicon sequencing variants (ASVs) corresponding to potential prey were assigned using the following criteria to taxonomical categories: ASVs with an identity higher than 97% were determined at the species level, ASVs between 93 and 97% were assigned to genus, ASVs between 93 and 90% were assigned to subfamily, and those below 90% were assigned to family or order^11^. Contamination reads were removed from downstream analyses to avoid confusion and included fungi, humans, vertebrates, insects, and some marine species analysed in the laboratory, like lobsters (see Appendix 1 for details).

### Multivariate analysis of the diet

The software PRIMER6 and PERMANOVA+^77^ was used to analyse the diet of *O. vulgaris* paralarvae. Relative read abundance (RRA) and frequency of observance (FOO) were used as input data, and resemblance matrices were obtained with Bray–Curtis and Jaccard distances, respectively. An unbiased representation of the prey detected in the multidimensional space was visualised using principal coordinate analysis (PCO) plots. Permutational multivariate analysis of variance (PERMANOVA) tests using 9999 permutations were run to test the factors described in Table 4. Factor ICC with two levels: NW Iberian Peninsula, and W Morocco. Factor location with three levels: C, coast for all the samples collected over the continental shelf (green colour in Fig. 7c,e); C~O, for the samples collected over the continental slope but under the influence of the upwelling filament or coastal upwelled water (orange colour in Fig. 7e); and O, ocean for all the samples collected in the open ocean (blue colour in Fig. 7c,e). Factor strata with three levels; < 5 for samples collected between 0 and 5 m; < 100, between 0 and 100 m; and < 500, between 0 and 500 m. Factor day/night with two levels. Finally, the factor suckers, with three levels according to the number of spa: 3 (n = 51), 4 (n = 31), and > 5 (n = 18). PERMANOVA analysis was performed using the ‘unrestricted permutation of raw data’ option for these independent factors. Furthermore, a two-factor PERMANOVA test was also performed for the factors' location and strata. The factor strata was nested within the different locations, using the “permutation of residuals under the full model”.

The prey contributing most to similarities and dissimilarities between the paralarvae collected at different locations of the ICC and the paralarvae grouped by sucker count was determined using the program SIMPER^78^, using the Bray–Curtis dissimilarity matrix and FOO as input data to avoid biases related with read number. SIMPER analysis allows the detection of the discriminant prey for the different factors analysed, their contribution to the total variability observed, and the discriminative power of the main prey driving the differences observed. Within each area of the ICC, the contribution of the different prey groups was analysed using FOO data with distance linear models (DistLM), applying an indicator that grouped the prey species at different taxonomic levels. A stepwise selection procedure was chosen using the adjusted R^2^ as the selection criterion for retaining prey groups that summarised the diets of the different paralarvae at the different places of the ICC sampled.

Two trophic indices were analysed to evaluate the behavioural changes in trophic selection: the linear index of food selection (L)^79^ for the different prey ingested and the trophic niche breadth (Czekanowski’s index, CI)^80^ for each paralarva. L ranges from − 1 to + 1, with positive values indicating a preference, negative values indicating avoidance or inaccessibility, and zero values showing random feeding^79^. This index was obtained for every prey detected with the formula (L = r_i_ − p_i_), where “r_i_” is the relative proportion of prey item i in the gut based on FOO and “p_i_” the relative proportion of prey item i in the zooplankton sample from which the paralarvae were selected. For this analysis, the mesozooplankton sample collected off the Ria de Vigo was considered separately from those of the NW Iberian Peninsula since they represent two zooplankton communities markedly different^9^. Prey were mainly grouped at the family level to unify the taxonomic levels obtained with the metagenomic study and the visual identification of the zooplankton. Furthermore, the trophic niche breadth for every octopus paralarvae was calculated using the CI^80^ to evaluate the degree of overlap between the prey and their distribution in the zooplankton. Trophic niche breadth was calculated with the formula (CI = 1 − 0.5 ∑i | r_i_ − p_i_ |), where r_i_ and p_i_ are the relative abundances of resource item i eaten by the paralarvae based on FOO data (ri) and in the zooplankton (pi) (Suppl. Table S2). Values of CI range from 1 for the broadest possible niche (a population uses resources in proportion to their availability as a generalist predator) to [min p_i_] for the narrowest possible niche (a population is specialised exclusively on the rarest resource). In the context of diet, we infer CI > 0.5 to reflect a generalist predator that targets prey in proportion to their availability, whereas CI < 0.5 reflects a specialist predator. Individual values of CI for each paralarvae were compared between the different ontogenetic groups (using spa as a factor) with PERMANOVA to test whether the foraging tactics of *O. vulgaris* changed with development.

## Supplementary Information

Supplementary Information 1.

Supplementary Information 2.

Supplementary Information.

## Data Availability

All data analysed during this study are included in this published article (and its Supplementary Information files). Raw files are available upon request.
