# Supplementary material for: Trophic ecology of Octopus vulgaris paralarvae along the Iberian Canary current eastern boundary upwelling system
Source: Sci Rep. 2023 May 30;13:8744. doi: 10.1038/s41598-023-35206-4 (PMC10229560; doi:10.1038/s41598-023-35206-4)
Supplement: Supplementary file 3 — Supplementary Information. [file 41598_2023_35206_MOESM3_ESM.docx]

**Trophic ecology of *Octopus vulgaris* paralarvae along the Iberian Canary current eastern boundary upwelling system**

**Supplementary Material**

The analyses shown in this study were done based on the filtered reads that are available in the excel supporting file named “Octopus vulgaris filtered reads”

**Table S1.** Summary of the prey reads detected in *Octopus vulgaris* paralarvae collected in two sub-regions along the ICC upwelling system (Northwest Iberian Peninsula and west Morocco). The details of the samples where the paralarvae were collected are shown at the top table: location (C, coast; C~O, samples collected in the upwelling filament and its periphery; and O, ocean); strata (<5, for samples collected between 0 and 5 m; <100, between 0 and 100 m; and <500, between 0 and 500 m); Day/Night (D/N); n, indicates the number of paralarvae analysed from each category, with superscripts showing the number of paralarvae where no prey DNA was detected; Sucker and Prey count, indicates the range of suckers present in the paralarvae as well as the total prey detected in the different categories. Within the prey table, the number of detections on different *O. vulgaris* paralarvae is shown (count), together with the total number of reads detected (reads) and their homology with public sequences (%). Colours represent prey detected in the coastal area (green), in the ocean (blue) or in both environments (light red).

**Table S2.** Relative abundance of the zooplankton groups at the stations where the diet of *Octopus vulgaris* paralarvae was analysed. Abbreviations as in Suppl. table S1. Station # as shown in Fig. 7.

**Table S3.** Primers used in this study. In italics: Illumina adapter sequence; I: inosine modification; bold: 8 bp index sequence for multiplexing

| **Primer name** | **Primer sequence (5’-to-3’)** |
| --- | --- |
| NICO-F | *TCGTCGGCAGCGTCAGATGTGTATAAGAGACAG*GGIACIGGITGAACWGTITAYCCHCC |
| NICO-R | *GTCTCGTGGGCTCGGAGATGTGTATAAGAGACAG*TAIACTTCIGGRTGICCRAARAAYCA |
| Amplicon_IDX-N701_1 | CAAGCAGAAGACGGCATACGAGAT**TCGCCTTA**GTCTCGTGGGCTCGG |
| Amplicon_IDX-N702_2 | CAAGCAGAAGACGGCATACGAGAT**CTAGTACG**GTCTCGTGGGCTCGG |
| Amplicon_IDX-N703_3 | CAAGCAGAAGACGGCATACGAGAT**TTCTGCCT**GTCTCGTGGGCTCGG |
| Amplicon_IDX-N704_4 | CAAGCAGAAGACGGCATACGAGAT**GCTCAGGA**GTCTCGTGGGCTCGG |
| Amplicon_IDX-N705_5 | CAAGCAGAAGACGGCATACGAGAT**AGGAGTCC**GTCTCGTGGGCTCGG |
| Amplicon_IDX-N706_6 | CAAGCAGAAGACGGCATACGAGAT**CATGCCTA**GTCTCGTGGGCTCGG |
| Amplicon_IDX-N707_7 | CAAGCAGAAGACGGCATACGAGAT**GTAGAGAG**GTCTCGTGGGCTCGG |
| Amplicon_IDX-N710_8 | CAAGCAGAAGACGGCATACGAGAT**CAGCCTCG**GTCTCGTGGGCTCGG |
| Amplicon_IDX-N711_9 | CAAGCAGAAGACGGCATACGAGAT**TGCCTCTT**GTCTCGTGGGCTCGG |
| Amplicon_IDX-N712_10 | CAAGCAGAAGACGGCATACGAGAT**TCCTCTAC**GTCTCGTGGGCTCGG |
| Amplicon_IDX-N714_11 | CAAGCAGAAGACGGCATACGAGAT**TCATGAGC**GTCTCGTGGGCTCGG |
| Amplicon_IDX-N715_12 | CAAGCAGAAGACGGCATACGAGAT**CCTGAGAT**GTCTCGTGGGCTCGG |
| Amplicon_IDX-N716_13 | CAAGCAGAAGACGGCATACGAGAT**TAGCGAGT**GTCTCGTGGGCTCGG |
| Amplicon_IDX-S502_A | AATGATACGGCGACCACCGAGATCTACAC**CTCTCTAT**TCGTCGGCAGCGTC |
| Amplicon_IDX-S503_B | AATGATACGGCGACCACCGAGATCTACAC**TATCCTCT**TCGTCGGCAGCGTC |
| Amplicon_IDX-S505_C | AATGATACGGCGACCACCGAGATCTACAC**GTAAGGAG**TCGTCGGCAGCGTC |
| Amplicon_IDX-S506_D | AATGATACGGCGACCACCGAGATCTACAC**ACTGCATA**TCGTCGGCAGCGTC |
| Amplicon_IDX-S507_E | AATGATACGGCGACCACCGAGATCTACAC**AAGGAGTA**TCGTCGGCAGCGTC |
| Amplicon_IDX-S508_F | AATGATACGGCGACCACCGAGATCTACAC**CTAAGCCT**TCGTCGGCAGCGTC |
| Amplicon_IDX-S510_G | AATGATACGGCGACCACCGAGATCTACAC**CGTCTAAT**TCGTCGGCAGCGTC |
| Amplicon_IDX-S511_H | AATGATACGGCGACCACCGAGATCTACAC**TCTCTCCG**TCGTCGGCAGCGTC |

**Fig. S1.** SIMPER analysis results showing the top discriminant species that account for >90% of the variability and define the different locations sampled in the two areas of the ICC (NW Iberian Peninsula and W Morocco) using different datasets: FOO, frequency of observance (%), Log (x+1) reads transformed with log (x+1) and RRA, relative read abundance (%). Left values indicate the explained variance of each species as percentage and the right values show their discriminative values in decreasing order.

**
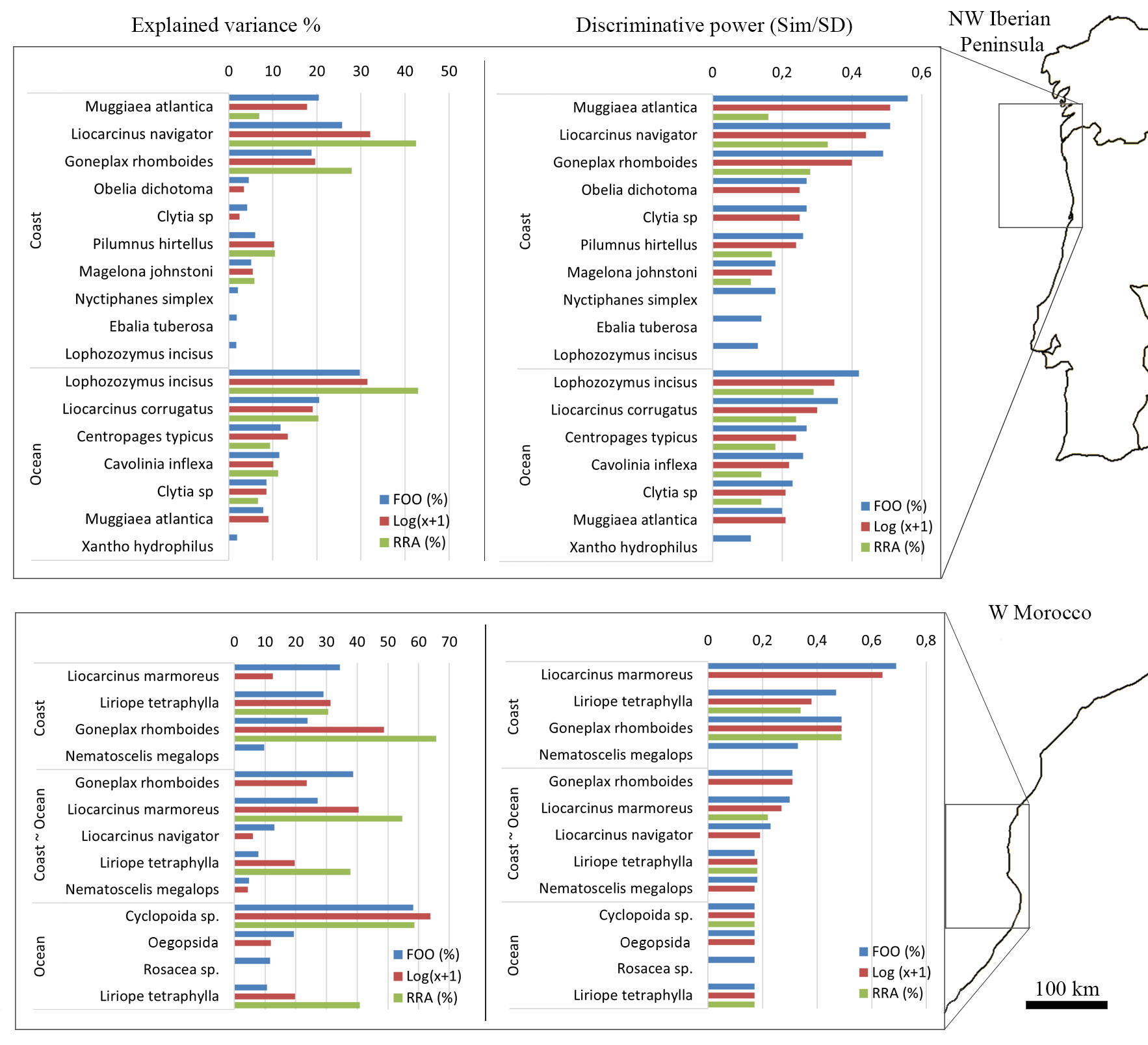
**
